# Supplementary material for: Intronic Alus Influence Alternative Splicing
Source: PLoS Genet. 2008 Sep 26;4(9):e1000204. doi: 10.1371/journal.pgen.1000204 (PMC2533698; doi:10.1371/journal.pgen.1000204)
Supplement: Figure S1 — Intron length distribution of human introns. (0.17 MB DOC) [file pgen.1000204.s001.doc]

**Figure S1: Intron length distribution of human introns.** In the x-axis the lengths of the introns in base pairs are shown whereas the y-axis presents the number of introns. The red line is found within 1000 bp (left to the red dashed line are introns smaller than 1000 bp and on the right the introns longer than 1000 bp).
