# Supplementary material for: Intronic Alus Influence Alternative Splicing
Source: PLoS Genet. 2008 Sep 26;4(9):e1000204. doi: 10.1371/journal.pgen.1000204 (PMC2533698; doi:10.1371/journal.pgen.1000204)
Supplement: Figure S2 — A screen shot created by the UCSC genome browser. (1.44 MB DOC) [file pgen.1000204.s002.doc]

**Figure S2: A screen shot created by the UCSC genome browser**, in which an exon is flanked by an Alu that undergoes editing, and by another adjacent Alu in opposite orientation. There is no closer opposite orientation Alu in the same intron, and hence the edited Alu potentially forms a dsRNA formation with the illustrated opposite orientation Alu.
