# Supplementary material for: Intronic Alus Influence Alternative Splicing
Source: PLoS Genet. 2008 Sep 26;4(9):e1000204. doi: 10.1371/journal.pgen.1000204 (PMC2533698; doi:10.1371/journal.pgen.1000204)

**Figure S3: Potential dsRNA of AluJo+ and AluSx-**. The potential dsRNA structure, as predicted by mFold, is illustrated.


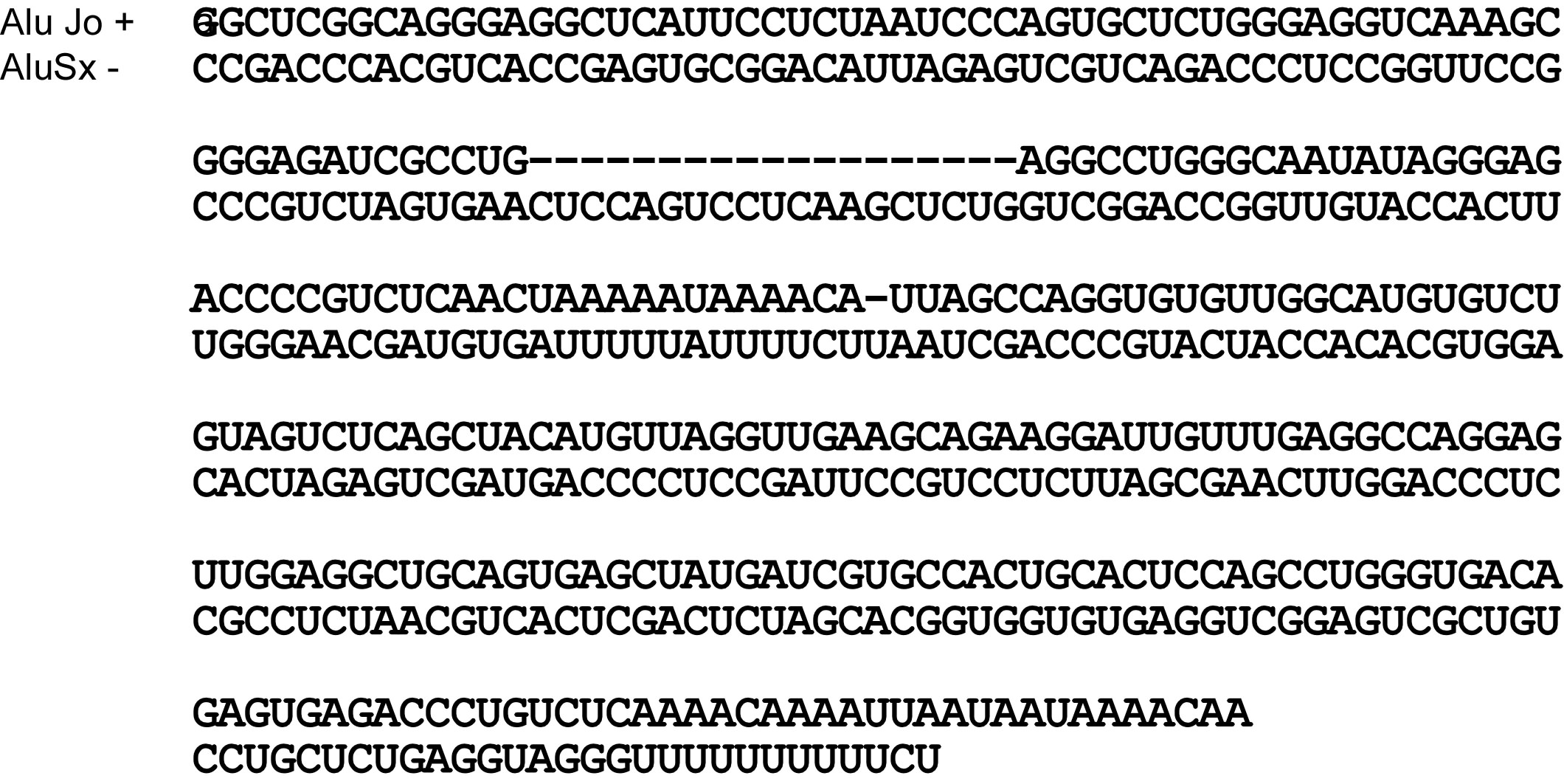

Supplement: Figure S3 — Potential dsRNA of AluJo+ and AluSx-. (0.51 MB DOC) [file pgen.1000204.s003.doc]
