# Supplementary material for: Intronic Alus Influence Alternative Splicing
Source: PLoS Genet. 2008 Sep 26;4(9):e1000204. doi: 10.1371/journal.pgen.1000204 (PMC2533698; doi:10.1371/journal.pgen.1000204)
Supplement: Text S1 — Minigenes' sequence. (0.03 MB DOC) [file pgen.1000204.s004.doc]

**Text S1:**

**Minigenes' sequence:**

Exons and introns sequences are in uppercase and lowercase, respectively. Sequences in red indicate the alternative exon. The intronic Alu sequences are highlighted in different coolers: Yellow, Red, Green, Blue, Pink, Blue, Gray represent sense AluJo, antisense AluSx, Left arm sense AluJo, sense AluSx, Right arm sense AluJo, antisense AluY, respectively.

**RABL5**

AGTGGAAAAACTGTTTTGGCCAACTTTCTGACAGAATCTTCTGACATCACTGAATACAGCCCAACCCAAGGAGTGAGgtgagccctgacaaatctgtgtcccagagtgtccaactggctcggcagggaggctcattcctctaatcccagtgctctgggaggtcaaagcgggaggatcgcctgaggcctgggcaatatagggagaccccgtctcaactaaaaataaaacattagccaggtgtgttggcatgtgtctgtagtctcagctacatgttaggttgaagcagaaggattgtttgaggccaggagttggaggctgcagtgagctatgatcgtgccactgcactccagcctgggtgacagagtgagaccctgtctcaaaacaaaattaataataaaacaattttaaaagactgcccagccatcacccacttcctacttttgttgctgttggcattccaaaagtgtaatgcagcctcattattggtgctgctactgttcgtcttttttttttgggatggagtctcgtcctgtcgctgaggctggagtgtggtggcacgatctcagctcactgcaatctccgcctcccaggttcaagcgattctcctgccttagcctccccagtagctgagatcacaggtgcacaccatcatgcccagctaattcttttatttttagtgtagcaagggtttcaccatgttggccaggctggtctcgaactcctgacctcaagtgatctgcccgccttggcctcccagactgctgagattacaggcgtgagccactgcacccagcctactgctcttctttttccttttagGATCCTAGAATTTGAGAACCCGCATGTTACCAGCAACAACAAAGGCACGGGCTGTGAATTCGAGCTATGGGACTGTGGTGGCGATGCTAAgtatgtttcctttaaagaaagtcacttcatcaaatggtttaaaaatcagctgcccaagccaggggcggtggctcacacctgtaattccagcactttgggaggccaaggtgggtggctcacctgaggagaagagttcaagagcagcctggccaacatggcgaaactccgtctctactaaaaatacaaaaaattagccaggcttggtggcaggcacatgtaatcccagttgggaggctgaggcaggagaactgcttgaacctggaggcggaggttgcagtgaaccctggaggttcaagcggaggttgcttgaacccgggaggcagaagttgcagtgagctgagatcgcaccattgtactccagcctgggcgacagagctggacttctagctcaaaaaacaaaacaaaacaaaaaaatcagctgcccaccaggcaccatgaagttgggcctagtatgtttcctggcacgtatgaggagcttgataaatgatttgctgacatgcattgctttatacactttagtgattaggttatgatgcagttagaagtcatcaaccttggccaggcgcagtggctgacgcctgtaatcccagtactttgggaggctgaggcaggcagatcacctcaggtcaggagttcaagaccagccggcccaacatgatgaaacctcattttaaaaaaaaaaaaaataggggccaggcaccatggctcacacctgtaatccattgctttgggagatcaccagagctcaggagtttgagaccagcctggccaacatagcgaaaccctgtctctactaaaaatacaaaacttagcccagtacggtggtgtgtaccagtaatcaggagactgaggcacgagaatcgcttgaacctgggaggtggacattgcggtgagccaagatcgtgctacggcactcaagcctgggcgacagagccagactcttgtctccaaaaataataataataataataatttttttttaatgaagaaatcatccacctttacaaaaaataaaattgacctgatgtggtcctagctactcaggaagctgaggcagaaggatcacttgggcccacattggaggctgcagtgagctatgatcccacaactgcacttcagcctgggagacagagcaagaccctgtctcttaaaaaaaaaaagtcatcccccaactttttttaaaattttttttatttttttgagacagagtctcgctctgtcacccacgctggagtgcagtggcacgatctcagctcactgcaagctctgcctcctgggttcacgccattctcctgcctcagcctcccgagtagctgggactataggtgcccacaaccacactcccctaacttttttgtatttttagtagagacggggtttcaccgtgttagccaggattgtctcgatctcctgacctcgtgatcgcccgcctcggcctcccaaagtgctgggattacaggtgtgagccaccacgcccagccgtcatagaccaacttttaatactaagatattctttttaaagtccacagttagaaaccagaatgacctaactttccctgttgttccttttcttcctcgcagGTTTGAGTCCTGCTGGCCGGCCCTGATGAAGGATGCTCATGGAGTGGTGATCGTCTTCAATGCTGACATCCCAAGCCACCGGAAGGAAATGGAGATGTGGTATTCCTGCTTTGTCCAACAGCCGTCCTTACAGGACACACAGTGTATGCTAATTGCACACCACAAACCAGGCTCTGGAGATGATAAAGGAAGCCTGTCTTTGT
