# Supplementary material for: Intronic Alus Influence Alternative Splicing
Source: PLoS Genet. 2008 Sep 26;4(9):e1000204. doi: 10.1371/journal.pgen.1000204 (PMC2533698; doi:10.1371/journal.pgen.1000204)
Supplement: Text S2 — 270 nucleotides non-Alu intronic sequence from intron 20 of IKBKAP gene. (0.02 MB DOC) [file pgen.1000204.s005.doc]

**Text S2**

**270 nucleotides non-Alu intronic sequence from intron 20 of IKBKAP gene:**

agaatcgtgacactcatcatataaaggagggcttctcttaacctgaggga

acacatgtgggttttaggtggcctgtgaacccagggagattgtacacacc

aaaccttgtctttgtgtatttattcaagtagaaagcccacagctttcaat

agatttacagcggggcctatgacccagaaaagcctgagctactcttgtga

aggaaatgactgattttctgaacctatttggaggaaactttgtattggaa

agatctatactaatgttttg
