# Supplementary material for: Intronic Alus Influence Alternative Splicing
Source: PLoS Genet. 2008 Sep 26;4(9):e1000204. doi: 10.1371/journal.pgen.1000204 (PMC2533698; doi:10.1371/journal.pgen.1000204)
Supplement: Text S3 — 800 nucleotides intronic sequence from intron 11 of IMP gene. (0.02 MB DOC) [file pgen.1000204.s006.doc]

**Text S3**

**800 nucleotides intronic sequence from intron 11 of IMP gene:**

atcactctgcactttctcccatattccccctactcatttgagaattctgt

gtgtcatatgagggacctttcccccatggaatcgaagtcctctttgtttt

tgatcttgcttggtccccatgggatggcttcagtgcttctgggttttcag

gctgggcaggggcagggttataacatagaaagagaacttaggttctaaca

caaactgatgggttcaaatccaagatccatctcatcaccccttcccccaa

tacactatatgatctgagcctcactttcctcgtcagtacaatattatgag

gctacgcagccaaagcccttcgctcacatactaacaccaagtaagtgtta

gttgtctgtcaccttccctggccaaggtccaggggagagagaagggagaa

tctgtcatagtgaaacaaggatgctgttagagtttcacaaacctaaatcc

ggttgctttagttgtcttaaaagctttgagcaaaaaccttgattgttctc

cagtggaggtgcagtcactgccctctatccgttggtcatttcatttgtgt

ccttgcctgttggcaagactccactgaaacctctctgggagattggtagg

tggagggggcaggaggccctacttagaaagtgtcattgaagccaatcctt

ctaactgaccacctctgccctcctaataattctggtgtgaaggcgtaatg

atgtgggcttcagggtctttgttcttcctcccctaagtcttcagaatggg

tagttgggagtaagggtggtagaaggggaactggatgaagtggacatggt
